# Identification of novel biomarker as citrullinated inter-alpha-trypsin inhibitor heavy chain 4, specifically increased in sera with experimental and rheumatoid arthritis

**DOI:** 10.1186/s13075-018-1562-7

**Published:** 2018-04-10

**Authors:** Hoshimi Kawaguchi, Isao Matsumoto, Atsumu Osada, Izumi Kurata, Hiroshi Ebe, Yuki Tanaka, Asuka Inoue, Naoto Umeda, Yuya Kondo, Hiroto Tsuboi, Yasuhiro Shinkai, Yoshito Kumagai, Akihito Ishigami, Takayuki Sumida

**Affiliations:** 1 Department of Internal Medicine, Faculty of Medicine, University of Tsukuba, 1-1-1 Tennodai, Tsukuba, 305-8575 Japan; 2 Environmental Biology Laboratory, Faculty of Medicine, University of Tsukuba, Tsukuba, Japan; 3 Molecular Regulation of Aging, Tokyo Metropolitan Institute of Gerontology, Tokyo, Japan

**Keywords:** Rheumatoid arthritis, Citrullinated proteins, Inter-alpha-trypsin inhibitor heavy chain 4, PAD

## Abstract

**Background:**

Anticitrullinated protein antibodies (ACPA) and citrullinated proteins play key roles in the pathogenesis of rheumatoid arthritis (RA). Many candidate citrullinated antigens have been identified in joints, but citrullinated proteins in sera are mostly uncertain in patients with RA. We explored the expression of citrullinated proteins in joints and sera of experimental arthritis, and we further investigated their specific expression correlated with the disease activity in patients with RA.

**Methods:**

Citrullinated protein expression in tissues was examined by IHC in peptide glucose-6-phosphate isomerase-induced arthritis (pGIA). Serum citrullinated proteins from pGIA were examined by Western blotting, and the sequence was identified by MS. With the same methods, serum citrullinated proteins were analyzed in patients with RA, primary Sjögren’s syndrome, systemic lupus erythematosus, and osteoarthritis as well as in healthy subjects, by Western blotting and MS. In patients with RA, the relationship between the expression of the identified protein (inter-alpha-trypsin inhibitor heavy chain 4 [ITIH4]) and clinical features was evaluated, and the levels of citrullinated ITIH4 were compared before and after biological treatment. The antibody response against citrullinated ITIH4 peptide was measured by enzyme-linked immunosorbent assay.

**Results:**

Citrullinated proteins were detected specifically in arthritic joints and sera from pGIA relative to controls. In sera, a common band of citrullinated protein at 120 kDa was revealed, and it fluctuated in parallel with arthritis score of pGIA by Western blotting. Interestingly, in 82% of RA patient sera, similar bands of citrullinated protein were specifically detected. These proteins were identified as citrullinated ITIH4, and especially the R438 site was commonly citrullinated between mice and humans. Citrullinated ITIH4 levels were associated with clinical parameters such as C-reactive protein (CRP), rheumatoid factor, and Disease Activity Score in 28 joints as measured by CRP in patients with RA. Its levels were decreased in correlation with the reduction of disease activity score after effective treatment in patients with RA. Moreover, antibody response to citrullinated epitope in ITIH4 was specifically observed in patients with RA.

**Conclusions:**

Our results suggest that serum citrullinated ITIH4 was specifically increased in patients with RA and could be a novel biomarker for assessing disease activity in patients with RA.

**Electronic supplementary material:**

The online version of this article (10.1186/s13075-018-1562-7) contains supplementary material, which is available to authorized users.

## Background

Rheumatoid arthritis (RA) is an autoimmune disease characterized by the inflammation and destruction of joints and surrounding tissues [1]. Most patients with RA are positive for anticitrullinated protein antibodies (ACPA), and these antibodies are highly specific for RA diagnosis [2]. ACPA appear years before the onset of clinical RA and are the predictive factor of joint radiographic progression [2]. In the mouse model, it was reported that ACPA appeared prior to the development of clinical disease in collagen-induced arthritis, and administration of anticitrullinated fibrinogen antibodies enhanced arthritis [3]. Thus, ACPA is considered to be involved in the pathogenesis of RA.

ACPA recognize various citrullinated proteins, such as vimentin, α-enolase, fibrinogen, fibronectin, and glucose-6-phosphate isomerase (GPI) [2, 4]. High levels of citrullinated proteins have been found in RA joints [5, 6]. Also, citrulline-specific T cells were increased in peripheral blood of patients with RA and decreased by therapy [7]. In murine models, immunization of citrullinated fibrinogen induced arthritis in HLA-DRB1*0401-transgenic mice, but unmodified fibrinogen did not [8]. However, we do not know what citrullinated antigens specifically revealed in joints and sera in arthritis.

GPI is a ubiquitous glycolytic enzyme and was identified as a pathogenic autoantigen in K/BxN arthritic mice [9]. Immunization with recombinant human GPI could induce arthritis in DBA/1 mice [10]. A major epitope of GPI-specific CD4^+^ T cells in glucose-6-phosphate isomerase-induced arthritis (GIA) was identified as peptide 325–339 glucose-6-phosphate isomerase (pGPI), and immunization with pGPI can also induce arthritis just as GIA does in DBA/1 mice (peptide 325–339 glucose-6-phosphate isomerase-induced arthritis [pGIA]) [11]. CD4^+^ T cells play a critical role in GIA [10, 12], and the effect of biological agents in GIA is similar to RA [12, 13]. However, the involvement of ACPA and citrullinated proteins have not investigated in this model.

In the present study, we explored the expression and commonality of citrullinated proteins in pGIA and patients with RA, and we further investigated its correlation with RA disease activity. We show the production of ACPA, and we detected citrullinated proteins in arthritic joints and sera in pGIA. Serum citrullinated protein in pGIA was identified as citrullinated inter-alpha-trypsin inhibitor heavy chain 4 (ITIH4). The expression of ITIH4 was increased in inflamed synovium at day 14 in pGIA, suggesting the association with arthritis. In addition, bands of the same size were also detected in sera of patients with RA by Western blotting, which were confirmed as citrullinated ITIH4 as well. The levels of citrullinated ITIH4 were specifically increased in sera of patients with RA and were clearly associated with RA disease activity. Moreover, antibody response to citrullinated epitope in ITIH4 was observed in patients with RA. Citrullinated ITIH4 could be a novel specific biomarker representing the disease activity of patients with RA.

## Methods

### Mice

Male DBA/1 mice were purchased from Charles River Japan (Tokyo, Japan), and used at 6–10 weeks of age. All mice were maintained under specific pathogen-free conditions at the University of Tsukuba. All experimental protocols were approved by the Institutional Animal Care and Use Committee of the University of Tsukuba, and all animal experiments were conducted in accordance with institutional ethics guidelines. All surgeries were performed under isoflurane anesthesia, and utmost care was taken to minimize suffering.

### Serum samples

Serum samples were collected from Japanese patients with RA (*n* = 60, mean age 52.2 years, range 20–73 years, females 80%) diagnosed by rheumatologists according to the 1987 American College of Rheumatology (ACR) classification criteria [14] or the 2010 ACR/European League Against Rheumatism classification criteria [15]. Serum samples of the disease control subjects were collected from Japanese patients with primary Sjögren’s syndrome (SS) (*n* = 27, mean age 58.8 years, range 26–80 years, females 96%), systemic lupus erythematosus (SLE) (*n* = 15, mean age 33.9 years, range 16–55 years, females 80%), or osteoarthritis (OA) (*n* = 12, mean age 56.3 years, range 37–80 years, females 67%). All the patients with SS were diagnosed by rheumatologists according to the 1999 Japanese Ministry of Health criteria for diagnosis of SS [16]. All the patients with SLE fulfilled the 1997 ACR classification criteria [17]. None of the patients with SS or SLE had overlapping RA. Serum samples were collected from healthy subjects (HS) (*n* = 30, mean age 49.0 years, range 34–65 years, females 80%). Serum samples were also collected from 17 patients with RA before and 24 weeks after treatment with biologic drugs (infliximab, *n* = 9; abatacept, *n* = 8). All samples were collected at the University of Tsukuba Hospital after informed consent was obtained. This study was reviewed and approved by the ethics committee of the University of Tsukuba.

### Peptide GPI-induced arthritis

DBA/1 mice were immunized with 25 μg of pGPI (Invitrogen/Thermo Fisher Scientific, Carlsbad, CA, USA) in complete Freund’s adjuvant (CFA) (BD Biosciences, San Jose, CA, USA). pGPI was emulsified with CFA at a 1:1 ratio (vol/vol), or PBS + CFA was prepared as a vehicle control. For induction of arthritis, 150 μl of the emulsion was injected intradermally at the base of the tails of the mice. Each mouse was also given an injection of 200 ng of pertussis toxin (Sigma-Aldrich, St. Louis, MO, USA) intraperitoneally on days 0 and 2 after immunization to induce arthritis. Arthritis was assessed every other day and evaluated using a scale of 0–3 for swelling and redness of each paw. The clinical score was the sum of the scores for four paws, as described previously [11].

### Measurement of anti-CCP antibodies in pGIA

Sera were obtained on days 0–28 every week from mice immunized with pGPI or control, and antibodies were measured by enzyme-linked immunosorbent assay (ELISA). Sera were diluted 1:25 in dilution buffer and added to the 96-well plate (Immunoscan CCPlus test kit; Euro Diagnostica, Malmö, Sweden) for 1 h at room temperature. After a washing step, horseradish peroxidase (HRP)-conjugated polyclonal rabbit antimouse immunoglobulin (Dako, Carpinteria, CA, USA) diluted 1:1000 was added for 30 minutes at room temperature. After another washing step, color was developed with 3,3′,5,5′-tetramethylbenzidine (TMB) microwell peroxidase substrate (KPL/SeraCare, Milford, MA, USA). The optical density (OD) was measured at 450 nm by using a microplate reader.

A standard pool was obtained by mixing sera obtained from several mice on day 28. The concentrations of antibodies in this pool were considered 100 U/ml. A standard curve was obtained using serum dilutions, and the Michaelis-Menten equation was used to convert OD values into units, as described previously [18].

### Measurement of anti-ITIH4 antibodies in patients with RA

Native peptide ITIH4_428–447_ and R438 citrullinated peptide ITIH4_428–447_ were synthesized (serum, purity 95%) and used in ELISA. Ninety-six-well plates (Nunc MaxiSorp; Thermo Fisher Scientific) were coated with 10 μg/ml peptides for 12 h at 4 °C. After washing and blocking steps, sera from patients with RA (*n* = 60) and HS (*n* = 30) were diluted 1:200 in 1% bovine serum albumin (BSA) in PBS, then added for 2 h at room temperature. After a washing step, HRP-conjugated goat antihuman immunoglobulin G (IgG) (heavy and light chains [H + L]) (Abcam, Cambridge, MA, USA) diluted 1:10,000 was added for 1 h at room temperature. After washing, color was developed with TMB microwell peroxidase substrate (KPL/SeraCare). The OD was measured at 450 nm by using a microplate reader. The cutoff value was determined as the mean + 2 SD of HS.

### Histopathological analysis

Ankle joint tissue samples were harvested at days 0 and 14 from mice immunized with pGPI or control. They were fixed in neutralized 10% formalin, embedded in paraffin, and sectioned. To detect citrullinated proteins, we prepared modification buffer by mixing Reagent A (20% H_2_SO_4_, 25% H_3_PO_4_, and 0.025% FeCl_3_) and Reagent B (1% diacetyl monoxime, 0.5% antipyrine, 1 M acetic acid) at a 2:1 ratio (vol/vol). The sections were covered with the modification buffer and incubated in a light-proof container at 37 °C for 2.5 h to modify citrulline residues. Then, the sections were incubated overnight at room temperature with rabbit anti-modified citrulline (AMC) polyclonal antibodies diluted 1:3200 in 2% BSA in PBS to detect modified citrulline residues [19]. The sections were also incubated with HRP-conjugated goat antirabbit IgG (H + L) (Bio-Rad Laboratories, Hercules, CA, USA) for 30 minutes at room temperature. The sections were also stained with 3,3′-diaminobenzidine (DAB) (Nichirei Biosciences, Tokyo, Japan) and hematoxylin. To detect ITIH4 or macrophages, IHC analysis of the joint or liver (as a positive control of ITIH4 staining) sections was performed as described above, using rabbit antihuman ITIH4 antibodies (Abcam) diluted 1:1000 and rat antimouse F4/80 antibodies (BioLegend, San Diego, CA, USA) diluted 1:200 as primary antibodies and using rabbit-specific IHC polymer detection kit HRP/DAB (Abcam) and HRP-conjugated polyclonal rabbit antirat immunoglobulin (Dako) diluted 1:200 as secondary antibodies, respectively.

### Real-time qPCR analysis

We extracted total RNA from the ankle joints of pGIA or control mice using the ISOGEN (Wako Pure Chemical Industries, Tokyo, Japan) extraction method according to the instructions provided by the manufacturer. The extracted RNA was reverse-transcribed to complementary DNA with random primers. We performed real-time qPCR using a TaqMan gene expression assay (Applied Biosystems/Thermo Fisher Scientific, Foster City, CA, USA), and the Padi4 (Mm01341658_m1) and glyceraldehyde 3-phosphate dehydrogenase (GAPDH) (NM_002046) primers. Real-time qPCR was carried out using an ABI 7500 analyzer (Applied Biosystems). The expression of GAPDH was used as the control.

### Western blot analysis

Serum samples were obtained at days 0, 7, 14, and 28 from mice immunized with pGPI or control, as well as from patients with RA, SS, SLE, or OA and from HS. For analysis of citrullinated protein expression, serum samples were loaded into each well (50 μg for SDS-PAGE or 100 μg for two-dimensional PAGE [2D-PAGE]), separated by SDS-PAGE or 2D-PAGE, and transferred to polyvinylidene difluoride membranes. The modification buffer was added to the blots before incubation in a light-proof container at 37 °C for 2.5 h to modify citrulline residues, as described above. Blots were washed with 0.05% Tween 20 in Tris-buffered saline (TBST) and blocked with 5% milk in TBST, then incubated overnight at 4 °C with AMC antibodies diluted 1:3200 in 5% milk in TBST [19]. After a washing step, the blots were incubated with secondary antibody HRP-conjugated goat antirabbit IgG (H + L) (Bio-Rad Laboratories) diluted 1:5000 in 5% milk in TBST for 1 h at room temperature. Densitometric analysis was carried out using an ImageQuant LAS 4000 densitometer (GE Healthcare Life Sciences, Marlborough, MA, USA). The band intensity was determined with ImageQuant TL software (GE Healthcare Life Sciences) and normalized using the value of one sample (pGIA or RA) as 10. The difference of citrullinated ITIH4 levels from baseline to 24 weeks after treatment was normalized using each value at baseline. For analysis of ITIH4 expression, serum samples were loaded 10 μg/well and Western blot analysis was performed as described above, using rabbit antihuman ITIH4 antibodies (Abcam) diluted 1:3000.

### Measurement of citrulline content

Sera were obtained at days 0, 7, 14, and 28 from mice immunized with pGPI or control, and citrulline contents were measured by using the color development reagent (COLDER) assay, as described previously [20]. Sera were diluted 1:100 in COLDER buffer (50 mM NaCl, 10 mM CaCl_2_, 2 mM dithiothreitol, and 100 mM Tris, pH 7.4), and 60 μl of samples were added to the 96-well plates (Nunc MaxiSorp). Then, 200 μl of COLDER were added to detect citrullinated proteins. The samples were incubated for 30 minutes at 95 °C. The OD was measured at 570 nm by using a microplate reader and compared with a standard curve of known citrulline concentrations. Data were normalized relative to the protein concentration in each sample.

### LC-MS

Sera from pGIA mice, control mice, patients with RA, and HS were separated by 2D-PAGE, and gels were stained with GelCode Blue Stain Reagent (Thermo Fisher Scientific) for Coomassie brilliant blue staining. Gel slices were incubated overnight with MS-grade modified trypsin (6.7 ng/μl; Promega, Madison, WI, USA) at 37 °C. The resultant peptides were analyzed with the nanoACQUITY ultrahigh-performance LC (UPLC) system (Waters, Milford, MA, USA). Data were collected in centroid mode from mass-to-charge ratios (*m/z*) 50 to 1990. All analyses were acquired with an independent reference. BiopharmLynx version 1.2 software (Waters) was used for baseline subtraction and smoothing, de-isotoping, de novo peptide sequence identification, and database searches.

### Statistical analysis

All data were expressed as mean ± SEM. Differences between groups were evaluated for statistical significance using Student’s *t* test. The Kruskal-Wallis test was used for evaluation of band intensity among the five subject groups. *P* values less than 0.05 were considered significant. Statistical analyses were performed using IBM SPSS Statistics software (IBM, Armonk, NY, USA).

## Results

### Overexpression of ACPA and citrullinated proteins in joints and sera in pGIA

First, we investigated the production of ACPA in pGIA. DBA/1 mice were immunized with pGPI, and we induced symmetrical polyarthritis characterized by severe swelling of the limb joints (Additional file 1: Figure S1a). ACPA titers were measured in pGIA sera by ELISA, and they were significantly higher than those of control mice (Additional file 1: Figure S1b).

**Fig. 1:**
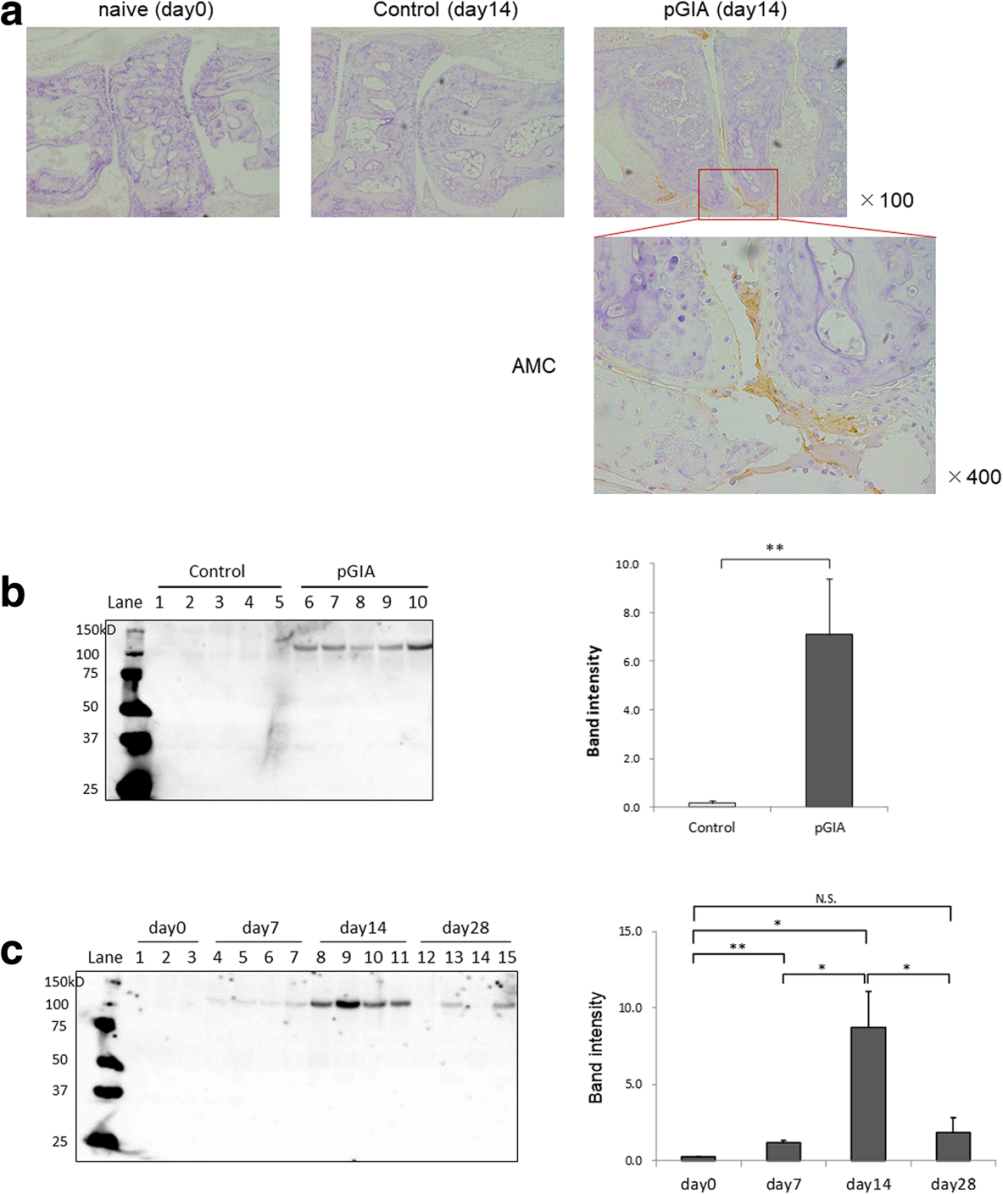
The increase of citrullinated proteins in arthritic joints and sera in peptide glucose-6-phosphate isomerase-induced arthritis (pGIA). **a** Joint sections day 0 and day 14 were immunohistochemically stained with anti-modified citrulline (AMC) antibodies in pGIA or control mice. Citrullinated proteins were detected at day 14 in pGIA mice (indicated by *boxed area*) but not in control mice. The bottom right image is a magnified view of the boxed area in the joint image at day 14 of pGIA. Original magnification × 100 and × 400 as marked. The experiments were performed on at least three different samples with similar results. **b** Sera from pGIA and control mice at day 14 subjected to Western blot analysis using AMC antibodies. Citrullinated proteins were detected as a common band at ~ 120 kDa in sera from pGIA mice but not from control mice. *Lanes 1–5*: control mice; *lanes 6–10*: pGIA mice. Representative blots are shown at left; data at right are mean ± SEM of band intensity (*n* = 9). **p < 0.01. **c** Sera from pGIA mice subjected to Western blot analysis. Citrullinated proteins appeared from day 7, increased at day 14, and decreased at day 28. *Lanes 1–3*: day 0; *lanes 4–7*: day 7, *lanes 8–11*: day 14, *lanes 12–15*: day 28. Representative blots are shown at left; data at right are mean ± SEM of band intensity (*n* = 4). *p < 0.05, **p < 0.01

Then, we evaluated the expression of citrullinated proteins in pGIA because high titers of autoantibodies against citrullinated proteins were detected. Ankle joint sections were stained with AMC antibodies by IHC. Citrullinated proteins were detected in joints at day 14 (the peak arthritic phase) and expressed in areas of synovial hyperplasia in pGIA but not in controls (Fig. 1a). Citrullinated proteins were not expressed in several other tissues examined (e.g., lung, spleen, lymph node) (data not shown). We analyzed the gene expression of Padi4, which is an enzyme that catalyzes protein citrullination, in articular tissue samples from pGIA by qPCR. At day 14, Padi4 levels tended to be higher in the arthritic joints of pGIA mice than in control mice, albeit insignificantly (Additional file 2: Figure S2a).

In our previous study, PAD4 levels were increased in sera of patients with RA [21], so we next explored the expression of citrullinated proteins in sera. Western blot analysis of serum samples using AMC antibodies identified citrullinated proteins in pGIA mice but in none of the control mice (Fig. 1b). Interestingly, the band of citrullinated protein was detected as a common band at ~ 120 kDa. The band was detected from the prearthritic phase and with significantly stronger intensity in the peak arthritic phase, then it decreased in line with the self-limited arthritis (Fig. 1c). Whereas citrullinated proteins were detected in serum samples (Additional file 3: Figure S3a, right), they were not detected when Western blot analysis was performed without modification of citrulline residues (Additional file 3: Figure S3a, left), suggesting that the detected band was really specific for citrulline.

To further confirm the serum levels of citrullinated proteins, we measured citrulline contents in pGIA sera using the COLDER assay. Serum citrulline content tended to be higher in pGIA at day 14 than in the controls (Additional file 3: Figure S3b). These results suggest that citrullinated proteins appeared in joints and sera in association with arthritis in pGIA.

### Identification of citrullinated ITIH4 in sera and increase of ITIH4 in arthritic joints in pGIA

**Fig. 2:**
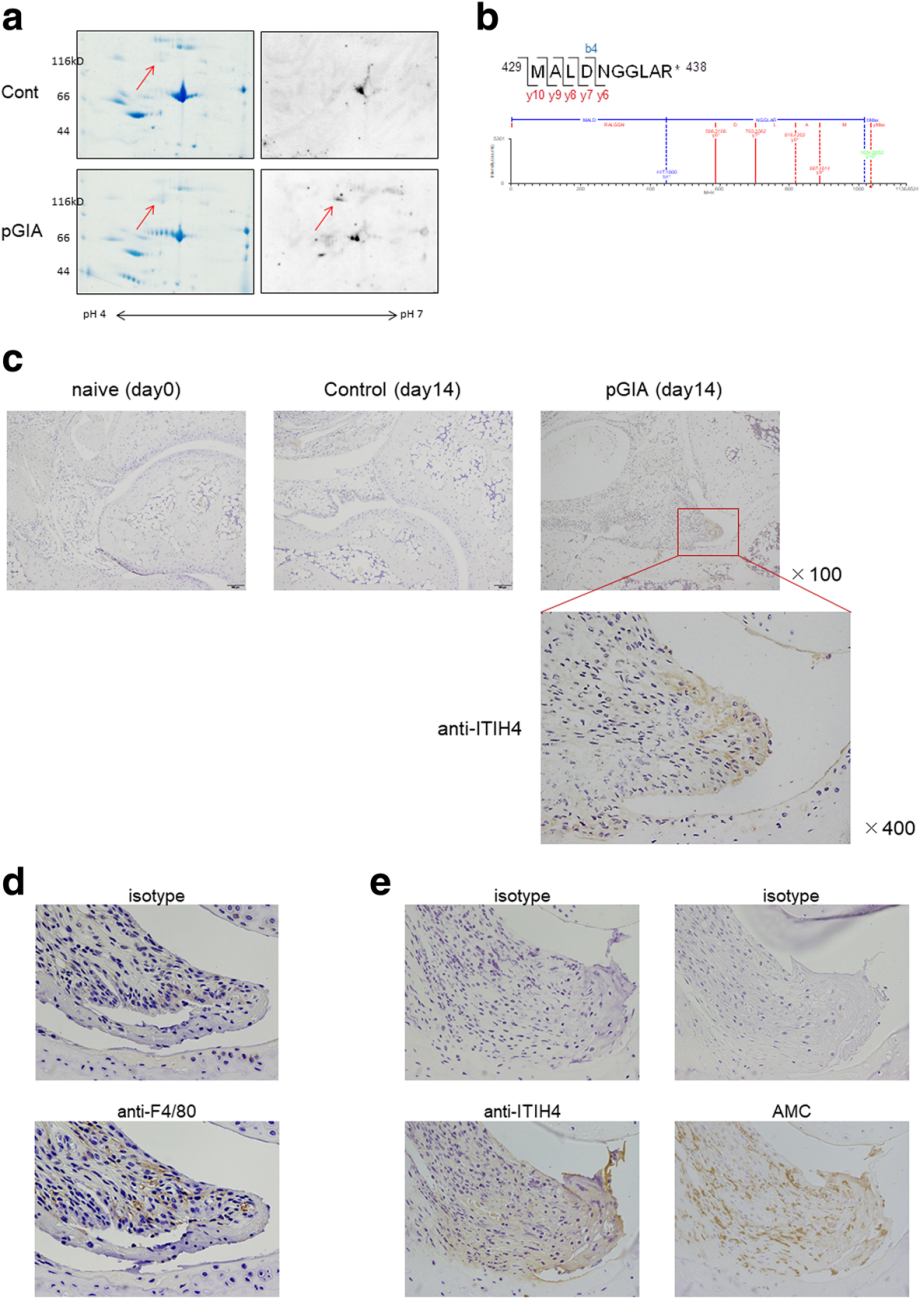
Identification of citrullinated inter-alpha-trypsin inhibitor heavy chain 4 (ITIH4) in sera and increase of ITIH4 in arthritic joints in peptide glucose-6-phosphate isomerase-induced arthritis (pGIA). **a** Serum samples from pGIA and control mice at day 14 separated by two-dimensional PAGE, then stained with Coomassie brilliant blue (left) or subjected to Western blot analysis using anti-modified citrulline (AMC) antibodies (right). Citrullinated proteins were detected at approximately 120 kDa and pH 5.3 in pGIA but not in controls. **b** The MS spectrum of ITIH4_429–438_ and modified peptides bearing the citrullinated arginine (R438) in pGIA. Citrullinated residues were identified by the unmodified b4 ion and the modified y6, y7, y8, y9, and y10 ion confirmed a mass increase of 1.0D. The experiments depicted in (**a**) and (**b**) were performed on at least three different samples with similar results. **c** Joint sections at day 0 and day 14 were immunohistochemically stained with anti-ITIH4 antibodies in pGIA or control mice. ITIH4 were detected at day 14 in pGIA (indicated by *boxed area*) but not in naïve or control mice at day 14. The image on the fourth column is a magnified view of the *boxed area* in the joint image at day 14 of pGIA. Original magnification × 100 and × 400 as marked. **d** Joint sections at day 14 were stained with anti-F4/80 antibodies for detection of macrophages (*bottom panel*) and isotype control antibodies (*top panel*) in pGIA. Macrophages colocalized with ITIH4 in some areas in inflamed synovium of pGIA. **e** Joint sections at day 14 were stained with anti-ITIH4 antibodies (*left bottom panel*), AMC antibodies (*right bottom panel*), and respective isotype control antibodies (*top panels*) in pGIA. ITIH4 colocalized with citrullinated proteins in inflamed synovium of pGIA in comparative serial sections. Original magnification × 400

To identify the 120 kDa citrullinated protein in sera, we analyzed the protein by MS. First, we separated the serum samples of pGIA by 2D-PAGE and confirmed the spot of citrullinated proteins at ~ 120 kDa by Western blot analysis (Fig. 2a, right). Second, the spot in the gels was sliced after Coomassie brilliant blue staining (Fig. 2a, left), digested with trypsin, and analyzed by nanoUPLC-MS to identify the protein. ITIH4 was identified with high coverage (Table 1), and we examined citrulline modifications by nanoUPLC-MS^E^. Citrullination of several sites containing R438 were identified in ITIH4 from pGIA (Table 1 and Fig. 2b). Analysis of the samples by MS after SDS-PAGE (Additional file 4: Figure S4a) also confirmed the presence of ITIH4 with high coverage, as well as citrullination at R438 in ITIH4 (Additional file 4: Figure S4b).

**Table 1:** Citrullinated inter-alpha-trypsin inhibitor heavy chain 4 was identified in mouse samples by two-dimensional PAGE and nanoUPLC-MS^E^

Protein ID	Protein name	Coverage (%)		MW (kDa)
		Control	pGIA	
O54882	Inter-alpha-trypsin inhibitor heavy chain 4	55.4	63.9	104.6
Position	Peptide sequence	Calculated mass	Observed mass	Arginine
429–438	MALDNGGLA(cit)	1033.50	1033.49	R438

*MW* Molecular weight, *pGIA* Peptide glucose-6-phosphate isomerase-induced arthritis

Calculated mass = calculated mass-to-charge ratio of the modified peptides; observed mass = observed mass-to-charge ratio of the modified peptides

To further investigate the association between sera and arthritic joints, we explored the expression of ITIH4 in joints in pGIA. Ankle joint sections were stained with anti-ITIH4 antibodies by IHC, and liver sections were stained as positive controls to detect ITIH4 (Additional file 5: Figure S5a). In naïve mice, ITIH4 was not detected in joints, but ITIH4 was clearly expressed in inflamed synovium at day 14 in pGIA (Fig. 2c). Macrophages detected by anti-F4/80 antibodies in serial sections showed colocalization with ITIH4 in some areas (Fig. 2d), suggesting that synovial macrophages may express ITIH4 in joints of pGIA. Moreover, ITIH4 depositions colocalized with citrullinated proteins in inflamed synovium in comparative serial sections (Fig. 2e), suggesting the presence of citrullinated ITIH4 in joints of pGIA. These results indicate the specific expression of ITIH4 and citrullinated ITIH4 in arthritic joints in pGIA.

### Specific increase of citrullinated ITIH4 in sera of patients with RA

**Fig. 3:**
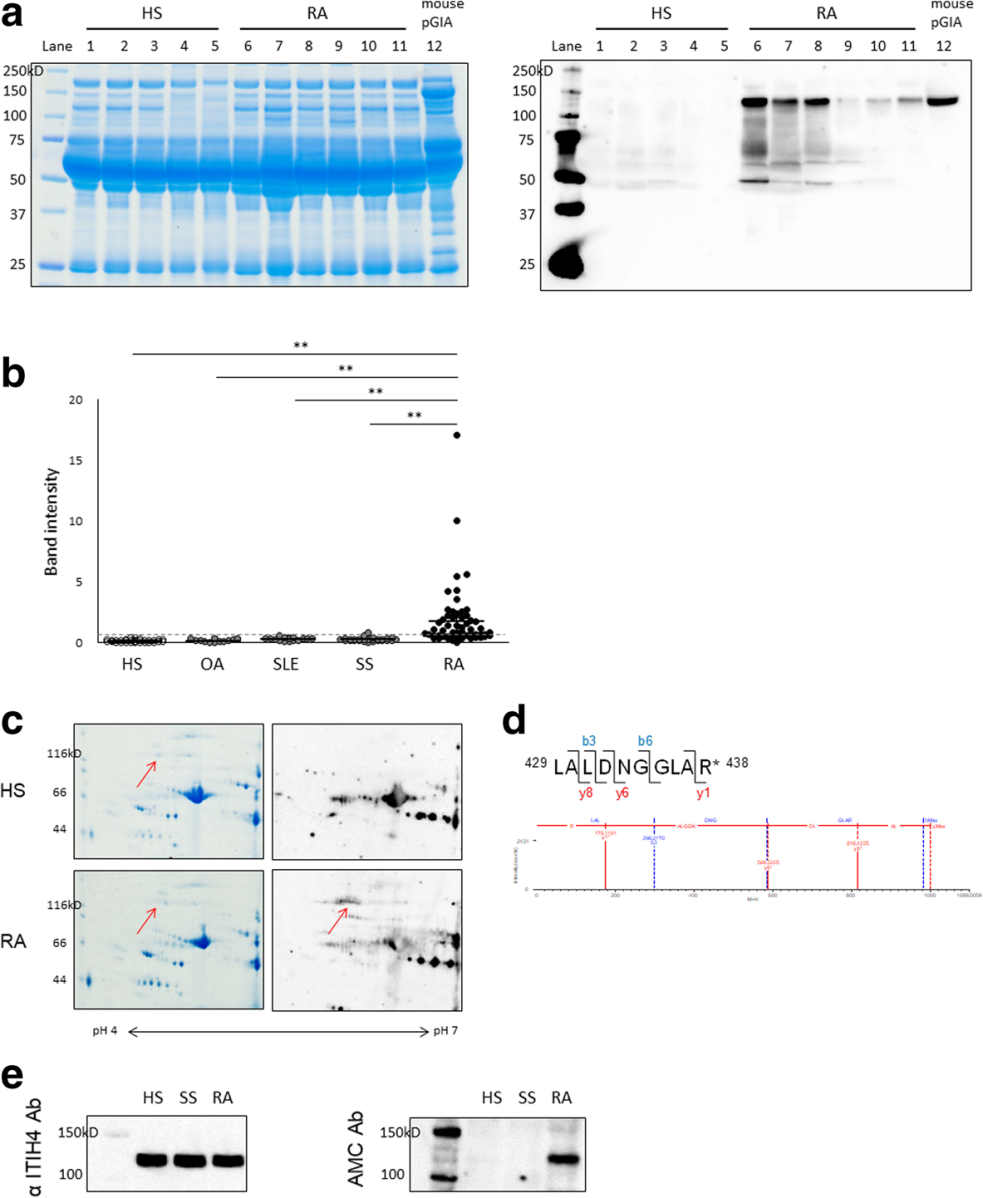
Specific increase of citrullinated inter-alpha-trypsin inhibitor heavy chain 4 (ITIH4) in sera in patients with rheumatoid arthritis (RA). **a** Sera were separated by SDS-PAGE from patients with RA, healthy subjects (HS), and peptide glucose-6-phosphate isomerase-induced arthritis (pGIA) mice, then stained with Coomassie brilliant blue (CBB) (*left*) or subjected to Western blot analysis using anti-modified citrulline (AMC) antibodies (*right*). Citrullinated proteins were detected as ~ 120 kDa bands in patients with RA and pGIA mice but in none of the HS. CBB staining is shown to demonstrate loading. *Lanes 1–5*: HS; *lanes 6–11*: RA; *lane 12*: pGIA mice at day 14. **b** The band intensity of 120 kDa citrullinated protein. Sera from patients with RA (*n* = 60), HS (*n* = 30), patients with osteoarthritis (OA) (*n* = 12), patients with systemic lupus erythematosus (SLE) (*n* = 15), and patients with Sjögren’s syndrome (SS) (*n* = 27) subjected to Western blot analysis using AMC antibodies. Citrullinated proteins at 120 kDa were specifically increased in patients with RA. Each symbol represents a single sample. Horizontal bars represent the mean values; vertical bars represent the SEM; and broken line represents the cutoff value. **p < 0.01, *N.S.* Not significant. **c** Serum samples from patients with RA and HS separated by two-dimensional PAGE, then stained with CBB (*left*) or subjected to Western blot analysis using AMC antibodies (*right*). Citrullinated proteins were detected at ~ 120 kDa and pH 5.3 in patients with RA but not in HS. **d** The MS spectrum of ITIH4_429–438_ and modified peptides bearing the citrullinated arginine (R438) in patients with RA. Citrullinated residues identified by the unmodified b3 and b6 ions and the modified y1, y6, and y8 ions confirmed a mass increase of 1.0 Da. The experiments shown in (**c**) and (**d**) were performed on at least three different samples with similar results. **e** Serum samples subjected to Western blot analysis using anti-ITIH4 antibodies (Ab) (*left*) and AMC antibodies (*right*). The bands were detected at 120 kDa by anti-ITIH4 antibodies as well as AMC antibodies

We also investigated the expression of citrullinated proteins in sera of 60 patients with RA and 30 HS, as well as in 12 patients with osteoarthritis (OA), 15 patients with SLE, and 27 patients with SS (disease controls). As well as in pGIA mice, the 120 kDa band of citrullinated protein was detected in 82% of patients with RA but in none of the control groups, except for being faintly detected in 2 patients with SS (7%) and 1 patient with SLE (7%) (Fig. 3a and Additional file 6: Figure S6a). The levels of this citrullinated protein were significantly higher in patients with RA than in controls (Fig. 3b).

We analyzed the 120 kDa citrullinated protein in patients with RA by 2D-PAGE and MS in the same way as pGIA mice. Using 2D-PAGE and Western blot analysis, we first confirmed the spot of citrullinated protein at ~ 120 kDa, similar to pGIA (Fig. 3c). Second, MS of this spot identified ITIH4 with high coverage (Table 2). Analysis of citrulline modifications identified citrullination of several sites in ITIH4 from patients with RA. Citrullinated sites varied among individual samples, but the citrullination of R438 was common among the examined samples (*n* = 3) (Table 2 and Fig. 3d).

**Table 2:** Citrullinated inter-alpha-trypsin inhibitor heavy chain 4 was identified in human samples by two-dimensional PAGE and nanoUPLC-MS^E^

Protein ID	Protein name	Coverage (%)		MW (kDa)
		HS	RA	
B7Z545	Inter-alpha-trypsin inhibitor heavy chain 4	40.0	–	99.7
B2RMS9	Inter-alpha-trypsin inhibitor heavy chain 4 (inter-alpha globulin inhibitor H4)	–	42.3	103.3
Position	Peptide sequence	Calculated mass	Observed mass	Arginine
429–438	LALDNGGLA(cit)	999.55	999.53	R438

*Abbreviations: HS* Healthy subjects, *MW* Molecular weight, *RA* Rheumatoid arthritis

Calculated mass = calculated mass-to-charge ratio of the modified peptides; observed mass = observed mass-to-charge ratio of the modified peptides

To confirm whether the 120 kDa citrullinated protein was ITIH4, we performed Western blot analysis of serum samples using anti-ITIH4 antibodies. The bands were detected at 120 kDa by anti-ITIH4 antibodies in the same position as the bands detected by AMC antibodies (Fig. 3e). In addition, the levels of total ITIH4 were similar in patients with RA and controls, whereas the levels of citrullinated ITIH4 were specifically increased in patients with RA relative to controls (Fig. 3e). Using ELISA, we also measured total ITIH4 concentrations in sera from 60 patients with RA and 30 HS who were the same individuals used in Western blot analysis. There was no significant difference in total ITIH4 levels between patients with RA and HS (data not shown). These results indicate that the levels of citrullinated ITIH4 rather than total ITIH4 were specifically increased in association with arthritis in sera of patients with RA.

### Correlation between citrullinated ITIH4 levels and disease activity in patients with RA

Citrullinated ITIH4 was previously identified as one of various citrullinated proteins in RA synovium [6]; however, its function and association with RA are not clear. We compared the clinical features of patients with RA with or without serum citrullinated ITIH4 as confirmed by Western blot analysis, using a band intensity cutoff value of 0.46, representing the mean + 3 SD of HS. C-reactive protein (CRP) and rheumatoid factor (RF) levels and Disease Activity Score in 28 joints measured by CRP (DAS28-CRP) scores were significantly higher in citrullinated ITIH4-positive than in ITIH4-negative patients (*p* = 0.047, *p* = 0.011, and *p* = 0.040, respectively), whereas there was no association between citrullinated ITIH4 and anti-CCP antibody titer or anti-CCP antibody-positive rate (Table 3). These results suggest that high levels of citrullinated ITIH4 were associated with inflammatory markers and disease activity of patients with RA.

**Table 3:** Comparison of clinical features of patients with rheumatoid arthritis with or without serum citrullinated inter-alpha-trypsin inhibitor heavy chain 4

	Total	Citrullinated ITIH4		*p* Value
		+	–	
No. of patients (%)	60	49 (82%)	11 (18%)	
Age, years	52.2 ± 1.9	51.3 ± 2.2	55.9 ± 3.9	0.355
Females, *n* (%)	48 (80%)	38 (78%)	10 (91%)	0.317
DAS28-CRP score	3.9 ± 0.2	4.1 ± 0.2	3.3 ± 0.4	0.040
CRP, mg/dl	2.18 ± 0.30	2.46 ± 0.34	0.93 ± 0.56	0.047
Anti-CCP antibodies, U/ml	131.6 ± 21.6	138.9 ± 24.6	93.4 ± 39.0	0.462
Positive, *n* (%)	46 (82%)	39 (83%)	7 (78%)	0.709
RF, U/ml	210.6 ± 46.1	242.3 ± 55.0	69.5 ± 35.5	0.011
MMP-3, ng/ml	198.5 ± 21.8	214.7 ± 25.7	127.9 ± 20.1	0.122
PSL use, *n* (%, mean dose in mg/day)	51 (85%, 6.9)	41 (84%, 7.1)	10 (91%, 6.4)	0.544
MTX use, *n* (%, mean dose in mg/week)	40 (67%, 10.1)	31 (63%, 10.1)	10 (91%, 10.0)	0.075

*Abbreviations: ITIH4* Inter-alpha-trypsin inhibitor heavy chain 4, *DAS28-CRP* Disease Activity Score in 28 joints as measured by C-reactive protein, *CRP* C-reactive protein, *CCP* Cyclic citrullinated peptide, *RF* Rheumatoid factor, *MMP-3* Matrix metalloproteinase 3, *PSL* Prednisolone, *MTX* Methotrexate

Values are mean ± SEM. The cutoff value for positive citrullinated ITIH4 was band intensity of 0.46, representing the mean + 3 SD of healthy subjects

In addition, we analyzed the relationship between serum citrullinated ITIH4 and RF/ACPA levels in patients with RA. The levels of citrullinated ITIH4 were significantly higher in RF^+^ ACPA^+^ patients compared with RF^−^ ACPA^+^, RF^−^ ACPA^−^ patients (p = 0.011, *p* = 0.025, respectively) (Table 4), suggesting that citrullinated ITIH4 levels were associated with RF levels in patients with RA. Interestingly, citrullinated ITIH4 were detected in 88% of RF^−^ ACPA^−^ patients, although those levels were low (Table 4). It was suggested that positivity of citrullinated ITIH4 could be a useful marker in seronegative patients with RA as well.

**Table 4:** Relationship between serum citrullinated inter-alpha-trypsin inhibitor heavy chain 4 and rheumatoid factor/anticitrullinated protein antibodies levels in patients with rheumatoid arthritis

	RF^+^ ACPA^+^	RF^+^ ACPA^−^	RF^−^ ACPA^+^	RF^−^ ACPA^−^
No. of patients (%)	42 (75%)	2 (4%)	4 (7%)	8 (14%)
Cit-ITIH4 level	2.2 ± 0.5	1.3 ± 0.9	0.6 ± 0.3	0.9 ± 0.3
Cit-ITIH4-positive, *n* (%)	38 (91%)	1 (50%)	1 (25%)	7 (88%)

*Abbreviations: Cit-ITIH4* Citrullinated inter-alpha-trypsin inhibitor heavy chain 4, *RF* Rheumatoid factor, *ACPA* Anticitrullinated protein antibody

Cit-ITIH4 levels are mean ± SEM of band intensity

**Fig. 4:**
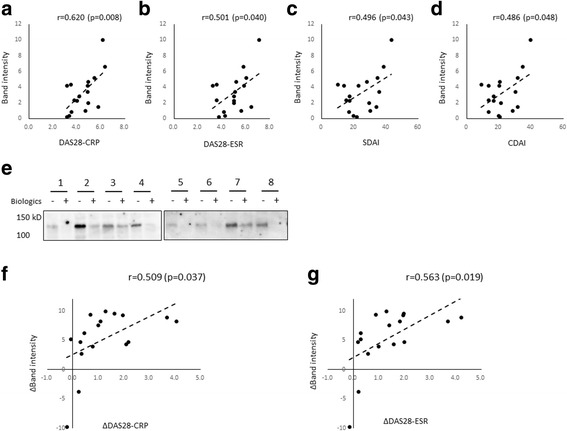
Relationship between citrullinated inter-alpha-trypsin inhibitor heavy chain 4 (ITIH4) levels and disease activity in patients with rheumatoid arthritis (RA). **a**–**d** Correlation between citrullinated ITIH4 levels and disease activity score in 28 joints (DAS28) as measured by C-reactive protein (DAS28-CRP) (**a**), DAS28-ESR (**b**), Simplified Disease Activity Index (SDAI) (**c**), and Clinical Disease Activity Index (CDAI) (**d**) at baseline in patients with RA (*n* = 17). **e** Sera from patients with RA at baseline or 24 weeks after biologic treatment subjected to Western blot analysis using anti-modified citrulline antibodies. Citrullinated ITIH4 levels were decreased after effective treatment. *Lanes 1–8*: Each individual, *lanes 1and 2*: abatacept treatment samples, *lanes 3–8*: infliximab treatment samples, Lanes marked (−): baseline samples; lanes marked (+):posttreatment samples. **f** and **g** Correlation between the difference of citrullinated ITIH4 levels (expressed as the normalized difference of band intensity) and the difference of (**f**) DAS28-CRP or (**g**) DAS28-ESR before and after treatment (*n* = 17). *r* is the correlation coefficient

To further clarify whether serum citrullinated ITIH4 levels were correlated with the disease activity of patients with RA, we recruited patients who continued biologic treatment for 24 weeks (infliximab, *n* = 9; abatacept, *n* = 8) among citrullinated ITIH4-positive patients. Because treatment with tocilizumab downregulates inflammatory markers sometimes irrespective of arthritis activity, we chose infliximab and abatacept in this experiment. At baseline, the levels of citrullinated ITIH4 were positively correlated with DAS28-CRP, DAS28-ESR, Simplified Disease Activity Index, and Clinical Disease Activity Index (Fig. 4a–d). In our comparison of the differences from baseline to 24 weeks after treatment, we found that citrullinated ITIH4 levels were decreased after treatment in responders (Fig. 4e). The difference of citrullinated ITIH4 levels was significantly correlated with the difference of DAS28-CRP and DAS28-ESR before and after treatment (Fig. 4f, g). These results suggest that serum citrullinated ITIH4 levels were positively correlated with disease severity and that citrullinated ITIH4 could be a serum marker reflecting disease activity in patients with RA.

### Autoantigenicity of citrullinated ITIH4

**Fig. 5:**
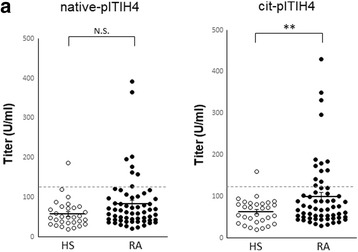
Autoantigenicity of citrullinated peptide 428–447 inter-alpha-trypsin inhibitor heavy chain 4 (pITIH4) in patients with rheumatoid arthritis (RA). **a** Antibody reactivities against native or citrullinated pITIH4 were analyzed in patients with RA (*n* = 60) and healthy subjects (HS) (*n* = 30) by enzyme-linked immunosorbent assay. Each symbol represents a single sample. Bars show the mean ± SEM; *broken line* represents the cutoff value. **p < 0.01. *N.S.* Not significant

Next, we investigated the autoantigenicity of citrullinated ITIH4. We synthesized a citrulline-containing ITIH4_428–447_ peptide (pITIH4) and the corresponding arginine-containing pITIH4 because R438 was identified as a dominant citrullination site. We assessed antibody reactivities against native or citrullinated pITIH4 by ELISA. On one hand, the levels of anticitrullinated pITIH4 antibody in patients with RA were significantly higher than those of HS (Fig. 5a, right). On the other hand, there was no significant difference in the levels of antinative pITIH4 antibody between patients with RA and HS (Fig. 5a, left). When the cutoff value was determined using sera from HS, 21.7% of RA sera was shown to be reactive with citrullinated pITIH4. The sensitivity and specificity were 21.7% and 96.7%, respectively. These results suggest the possibility of an autoimmune reaction to citrullinated ITIH4 in patients with RA.

## Discussion

To our knowledge, this is the first report on high levels of citrullinated ITIH4 in arthritis. ITIH4 is a heavy-chain protein, and ITIHs form inter-alpha-trypsin inhibitors (ITIs) by binding to a common light chain [22, 23]. Members of the ITI family are recruited to extravascular sites, where ITIHs are converted to hyaluronan (HA), to form serum-derived HA-associated protein (SHAP)-HA complexes in HA-rich tissues such as synovial fluid [22, 23]. Researchers in previous studies reported high levels of SHAP-HA complexes in RA serum and synovial fluid, suggesting their association with inflammation through the regulation of adhesion of infiltrating leukocytes [24]. In particular, ITIH4 was identified as one of various citrullinated proteins in RA synovium [6]. In addition, human ITIHs are reported to inhibit the complement system, suggesting a link to the immunocomplex cascades [25]. More recently, high serum levels of anticitrullinated ITIH3 antibodies were found in patients with RA [26].

In the present study, we show that citrullinated ITIH4 was recognized with high specificity in patients with RA as compared with patients with other autoimmune and arthritic diseases or in HS, indicating a potential role for citrullinated ITIH4 in RA pathogenesis. Additionally, in the investigation of autoantigenicity of citrullinated ITIH4, higher levels of anticitrullinated pITIH4 antibody were observed in patients with RA than in HS, whereas there was no significant difference in the levels of antinative pITIH4 antibody. These results suggest that citrullinated ITIH4 may be one of the candidate targets of ACPA and may be involved in the inflammatory response by forming immune complexes, which play an important role in the pathophysiology of RA [27]. ITIH4 has been reported to have an anti-inflammatory role, such as inhibition of the phagocytosis of polymorphonuclear cells [28]. Regarding the pathogenetic role of citrullinated ITIH4 in RA, another possibility is that citrullination of ITIH4 might suppress the anti-inflammatory effect of ITIH4 in situ, because the biologic activity of many proteins will be affected by their citrullination. Further studies are needed to evaluate the precise role of citrullinated ITIH4 in RA pathogenesis.

Which cells express ITIH4, and how and where is ITIH4 citrullinated? In this study, ITIH4 and macrophages colocalized in some areas in inflamed synovium of pGIA. These results suggest that synovial macrophages may express ITIH4 in pGIA, but there is a limitation of comparing serial sections by IHC. In addition, ITIH4 was also found in synovial or cartilage surface layers in pGIA, suggesting the presence of ITIH4 in the synovial fluid. It is possible that circulating ITIH4 was recruited into synovial fluid, because the ITI family was synthesized by hepatocytes and recruited to extravascular sites such as the synovial fluid [22, 23], and ITIH4 and citrullinated ITIH4 were also detected in RA synovial fluid (our preliminary observations), supporting the possibility of this scenario. In previous reports, peptidyl arginine deiminase 2 (PAD2) and PAD4 enzymes were increased in RA synovium or synovial fluid, and they contributed to protein citrullination in arthritic joints [5, 29, 30]. In this study, we show higher levels of Padi4 in the arthritic joints of pGIA, as well as the expression of ITIH4 and citrullinated ITIH4 in inflamed synovium of pGIA. Taking these findings together, we speculate that citrullination of ITIH4 was induced by aberrant PAD activity in arthritic joints and leaked into the blood circulation.

In this study, on one hand, serum citrullinated ITIH4 levels varied in relation to the clinical findings of pGIA, increasing at day 14 then falling at day 28. On the other hand, ACPA titers were significantly higher after day 21, despite the gradual resolution of arthritis. Furthermore, our preliminary studies showed that treatment with Cl-amidine (PAD inhibitor) resulted in suppression of arthritis coupled with a significant decrease in serum citrullinated ITIH4 levels, although there was no significant decrease in ACPA titers in pGIA. In patients with RA, the levels of CRP, RF, and DAS28-CRP were significantly higher in patients with RA positive for serum citrullinated ITIH4. In addition, basal serum citrullinated ITIH4 levels were significantly correlated with disease activity markers in patients with active RA and decreased after effective treatment in correlation with the disease activity. These results suggest that citrullinated ITIH4, not ACPA, seems to fluctuate in parallel with the severity of arthritis and could be a disease-specific biomarker representing the disease activity of patients with RA.

We observed that there were several citrullinated sites in ITIH4, and those sites were not identical between pGIA and patients with RA, and they were different between individuals. However, the R438 site was commonly citrullinated in our analyzed samples. Thus, this position is considered as a dominant citrullinated site. We show that the antibody reactivities against ITIH4_428–447_ peptides containing citrullinated R438 were significantly higher in patients with RA than in HS, whereas there was no significant difference in the reactivities against native ITIH4_428–447_ peptides containing arginine. Furthermore, the levels of anticitrullinated pITIH4 antibody tended to be higher than those of native pITIH4 in patients with RA, although there was no significant difference. There is a possibility that this citrullinated epitope in ITIH4 may be one of the targets of the immune system in RA. Nonetheless, the reactivities against the synthesized linear citrullinated peptides do not necessarily completely reflect the reactivities against the corresponding citrullinated proteins in vivo, and there is also a possibility of antibodies cross-reacting. It is necessary to verify in future studies the antibody reactivities against whole citrullinated ITIH4 or circulating citrullinated epitopes at several other sites.

## Conclusions

We show in the present study the specific expression of citrullinated proteins in joints and sera in pGIA. Notably, we demonstrate that the increase of specific citrullinated protein in sera as citrullinated ITIH4 fluctuated with the arthritis score. Also, in patients with RA, citrullinated ITIH4 levels were specifically increased in sera and significantly correlated with disease activity. Therefore, citrullinated ITIH4 could be a novel biomarker to distinguish RA from other rheumatic diseases and for assessing disease activity in patients with RA.

## Additional files

Additional file 1: **Figure S1.** DBA/1 mice were immunized with pGPI. (a) The clinical score (mean ± SEM) of pGIA (*n* = 11). (b) Sera were obtained once per week between days 0 and 28 from pGIA and control mice. The titers of anti-CCP antibodies were analyzed by ELISA (*n* = 12–18). Each symbol represents a single mouse, and the horizontal and vertical bars represent the mean and SEM values, respectively. *p < 0.05, **p < 0.01. (TIFF 120 kb)

Additional file 2: **Figure S2.** (a) *Padi4* gene expression levels in articular tissue samples from pGIA and control mice, analyzed by qPCR (*n* = 6). Data are mean ± SEM. (TIFF 97 kb)

Additional file 3: **Figure S3.** (a) Serum samples obtained at day 14 from pGIA mice. Western blot analysis was performed without modification of citrulline residues, but no bands of citrullinated proteins were detected (*left*). Western blot analysis performed with modification of citrulline residues allowed detection of the bands (*right*). *G1–G3* Samples of different mice. (b) Serum of mice immunized with pGPI or CFA control were subjected to the color development reagent assay to measure citrulline content (*n* = 6). Each symbol represents a single mouse. The horizontal and vertical bars represent the mean and SEM values for the group, respectively. (TIFF 149 kb)

Additional file 4: **Figure S4.** (a) Sera of pGIA and control mice obtained at day 14 were separated by SDS-PAGE and stained with Coomassie brilliant blue (*left*) or subjected to Western blot analysis using AMC antibodies (*right*). Citrullinated proteins were detected at ~ 120 kDa in pGIA mice but not in the control mice. G1, 2: pGIA; C1, 2: control mice. (b) The MS spectrum of ITIH4_429–438_ and modified peptides bearing the citrullinated arginine (R438) in pGIA. Citrullinated residues were identified by the modified y6, y7, and y8 ion confirmed a mass increase of 1.0 Da. (TIFF 217 kb)

Additional file 5: **Figure S5.** (a) Liver tissue sections from naïve mice were immunohistochemically stained with anti-ITIH4 antibodies as a positive control to detect ITIH4. (TIFF 263 kb)

Additional file 6: **Figure S6.** (a) Sera from patients with RA, HS, patients with OA, patients with SLE, and patients with SS was subjected to Western blot analysis using AMC antibodies. Citrullinated proteins were specifically detected as an ~ 120 kDa band in patients with RA. (TIFF 238 kb)

## Abbreviations

2D-PAGE: Two-dimensional PAGE
ABT: Abatacept
ACPA: Anticitrullinated protein antibodies
ACR: American College of Rheumatology
AMC antibodies: Anti-modified citrulline antibodies
BSA: Bovine serum albumin
CBB: Coomassie brilliant blue
CCP: Cyclic citrullinated peptide
CFA: Complete Freund’s adjuvant
COLDER: Color development reagent
CRP: C-reactive protein
DAB: 3,3′-Diaminobenzidine
DAS28: Disease Activity Score in 28 joints
ELISA: Enzyme-linked immunosorbent assay
ESR: Erythrocyte sedimentation rate
GAPDH: Glyceraldehyde 3-phosphate dehydrogenase
GIA: Glucose-6-phosphate isomerase-induced arthritis
GPI: Glucose-6-phosphate isomerase
HA: Hyaluronan
HRP: Horseradish peroxidase
HS: Healthy subjects
IFX: Infliximab
IgG: Immunoglobulin G
ITI: Inter-alpha-trypsin inhibitor
ITIH: Inter-alpha-trypsin inhibitor heavy chain
*m/z*: Mass-to-charge ratio
MMP-3: Matrix metalloproteinase 3
MTX: Methotrexate
MW: Molecular weight
OA: Osteoarthritis
OD: Optical density
PAD: Peptidyl arginine deiminase
pGIA: Peptide glucose-6-phosphate isomerase-induced arthritis
pGPI: Peptide 325–339 glucose-6-phosphate isomerase
PSL: Prednisolone
RA: Rheumatoid arthritis
RF: Rheumatoid factor
SDAI: Simplified Disease Activity Index
SHAP: Serum-derived hyaluronan-associated protein
SLE: Systemic lupus erythematosus
SS: Sjögren’s syndrome
TBST: Tween 20 in Tris-buffered saline
TMB: 3,3′,5,5′-Tetramethylbenzidine
UPLC: Ultrahigh-performance LC
