# Supplementary figures and images for: Identification of novel biomarker as citrullinated inter-alpha-trypsin inhibitor heavy chain 4, specifically increased in sera with experimental and rheumatoid arthritis
Source: Arthritis Res Ther. 2018 Apr 10;20:66. doi: 10.1186/s13075-018-1562-7 (PMC5894205; doi:10.1186/s13075-018-1562-7)

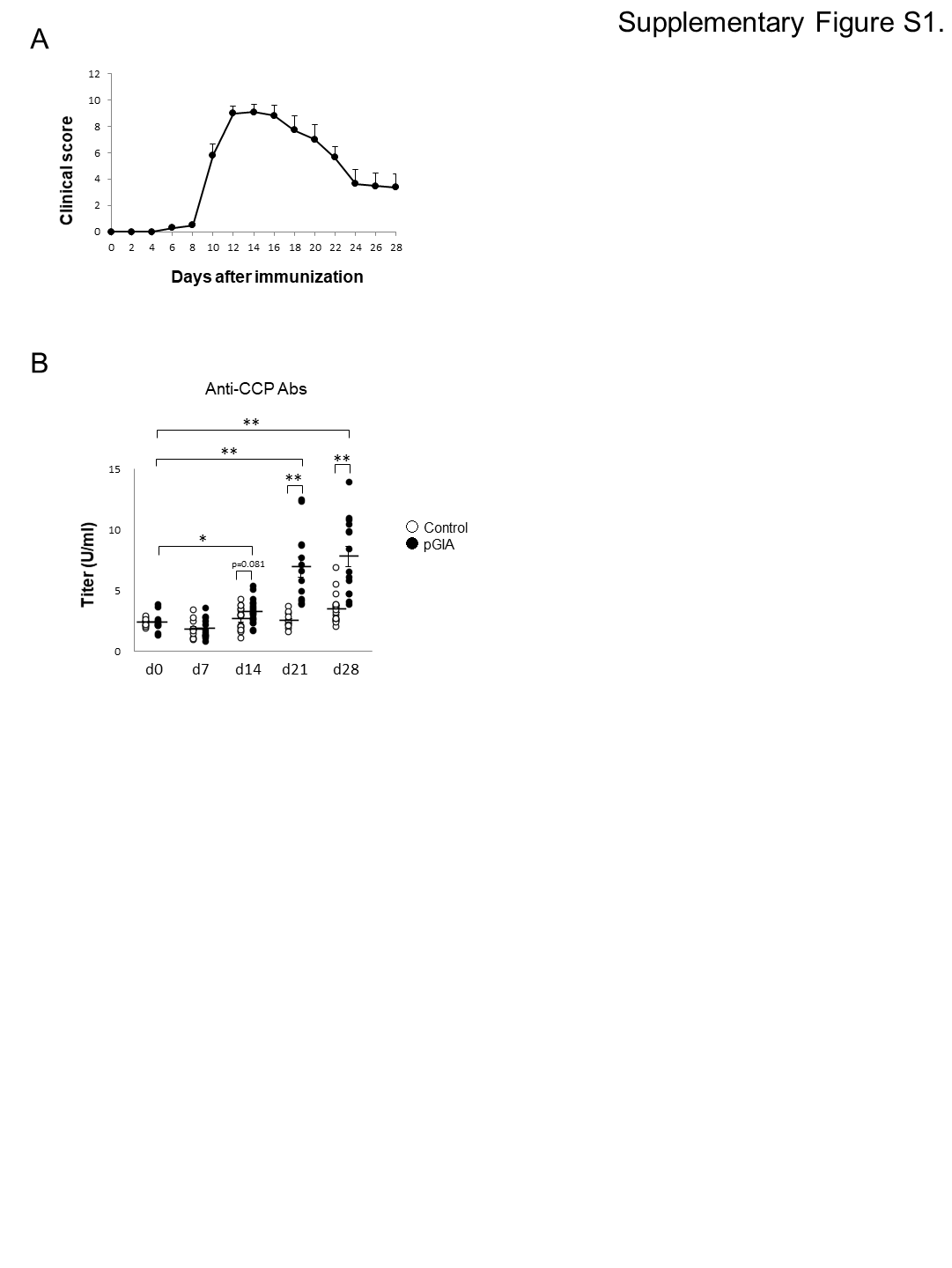

Supplement: Supplementary file 1 — Figure S1. DBA/1 mice were immunized with pGPI. (a) The clinical score (mean ± SEM) of pGIA (n = 11). (b) Sera were obtained once per week between days 0 and 28 from pGIA and control mice. The titers of anti-CCP antibodies were analyzed by ELISA (n = 12–18). Each symbol represents a single mouse, and the horizontal and vertical bars represent the mean and SEM values, respectively. *p < 0.05, **p < 0.01. (TIFF 120 kb) [file 13075_2018_1562_MOESM1_ESM.tif]

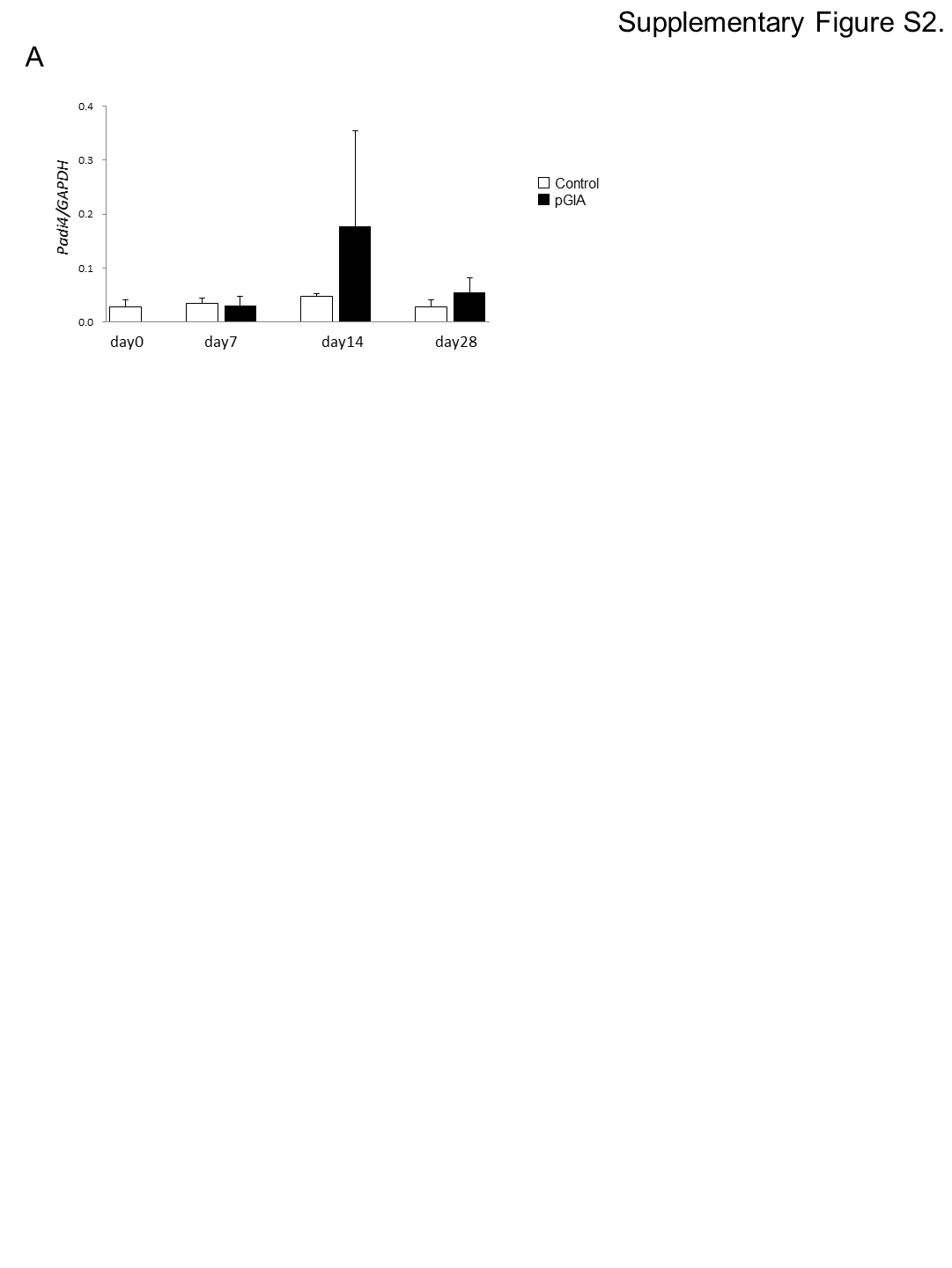

Supplement: Supplementary file 2 — Figure S2. (a) Padi4 gene expression levels in articular tissue samples from pGIA and control mice, analyzed by qPCR (n = 6). Data are mean ± SEM. (TIFF 97 kb) [file 13075_2018_1562_MOESM2_ESM.tif]

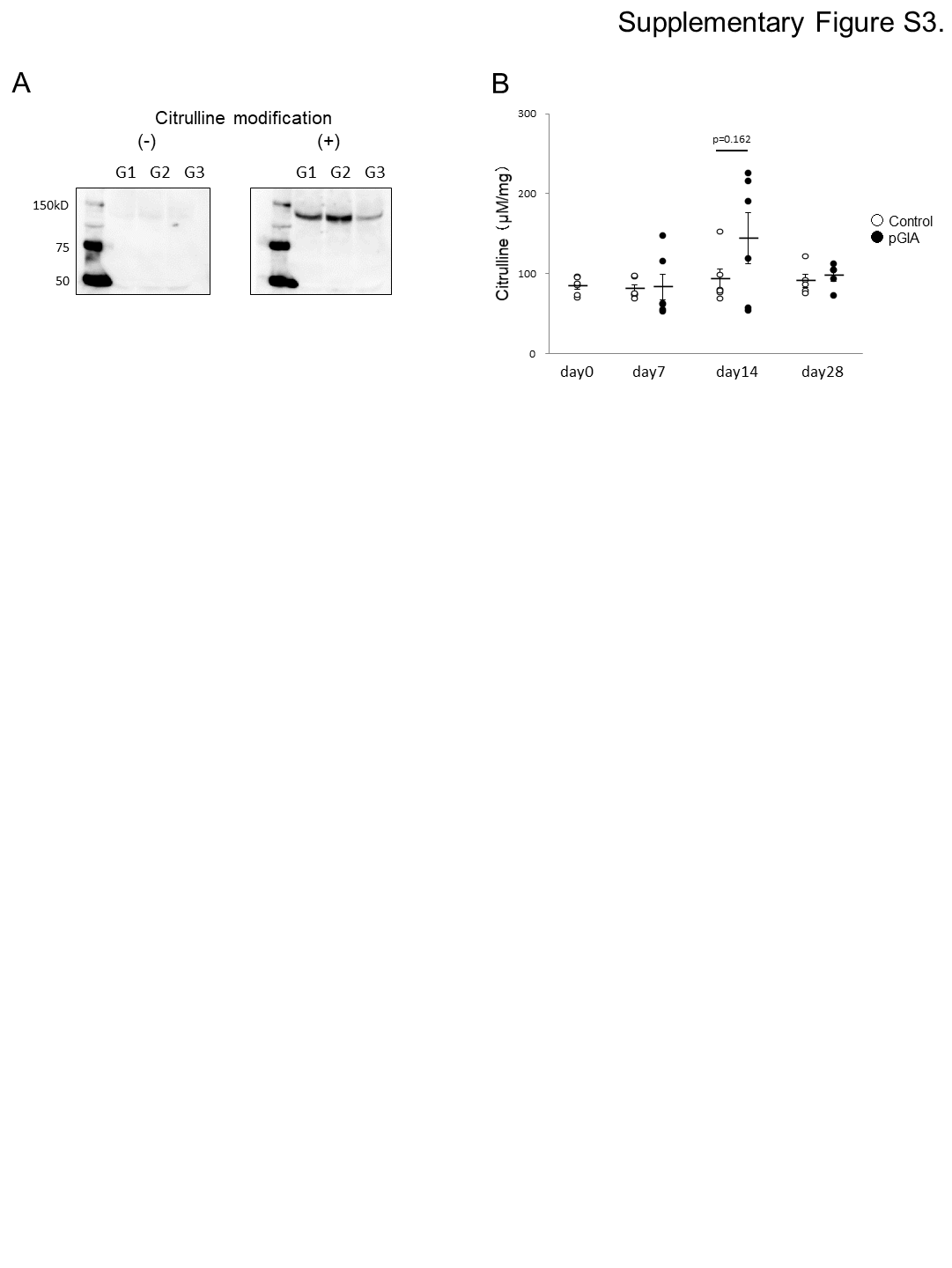

Supplement: Supplementary file 3 — Figure S3. (a) Serum samples obtained at day 14 from pGIA mice. Western blot analysis was performed without modification of citrulline residues, but no bands of citrullinated proteins were detected (left). Western blot analysis performed with modification of citrulline residues allowed detection of the bands (right). G1–G3 Samples of different mice. (b) Serum of mice immunized with pGPI or CFA control were subjected to the color development reagent assay to measure citrulline content (n = 6). Each symbol represents a single mouse. The horizontal and vertical bars represent the mean and SEM values for the group, respectively. (TIFF 149 kb) [file 13075_2018_1562_MOESM3_ESM.tif]

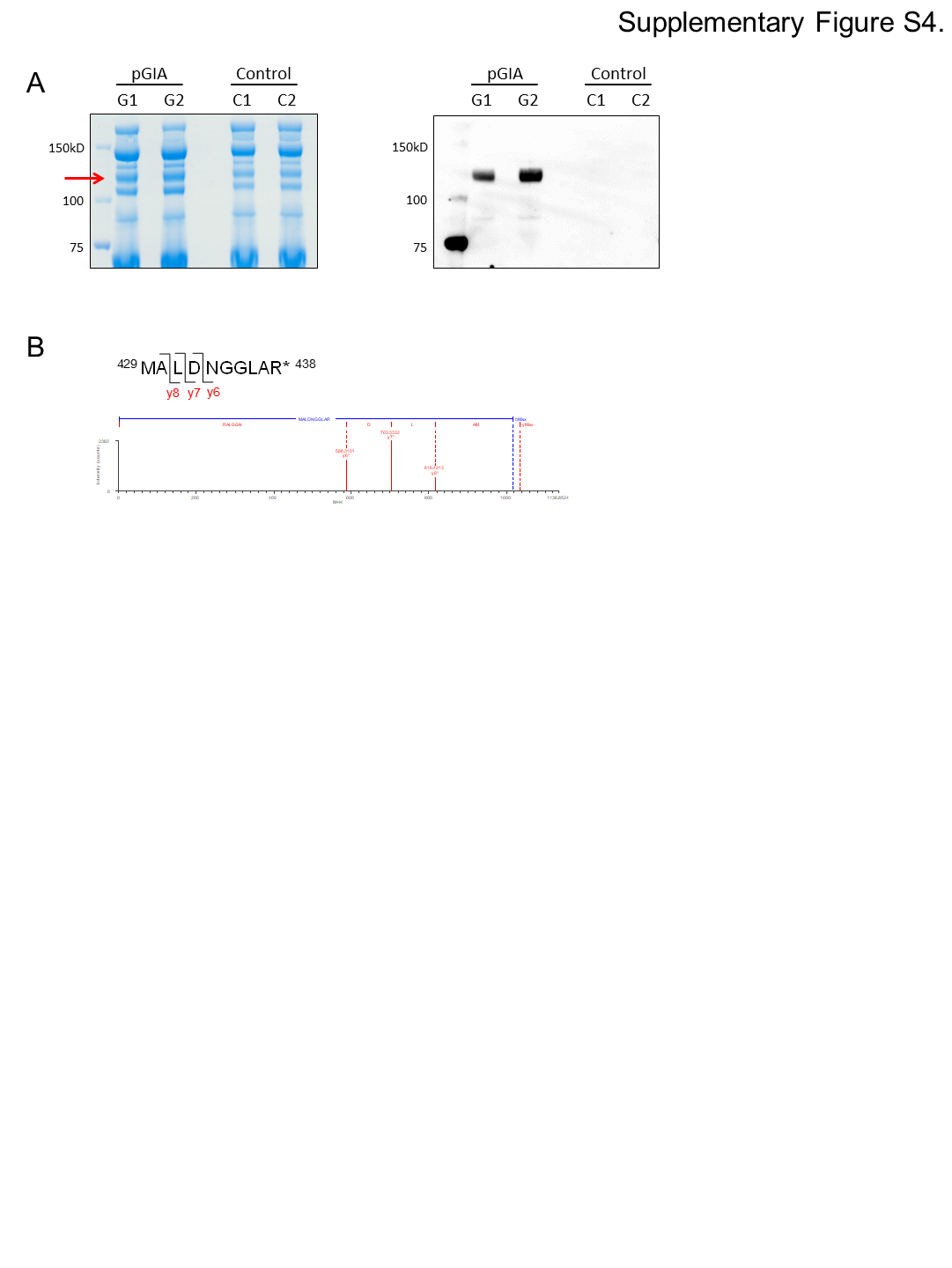

Supplement: Supplementary file 4 — Figure S4. (a) Sera of pGIA and control mice obtained at day 14 were separated by SDS-PAGE and stained with Coomassie brilliant blue (left) or subjected to Western blot analysis using AMC antibodies (right). Citrullinated proteins were detected at ~ 120 kDa in pGIA mice but not in the control mice. G1, 2: pGIA; C1, 2: control mice. (b) The MS spectrum of ITIH4429–438 and modified peptides bearing the citrullinated arginine (R438) in pGIA. Citrullinated residues were identified by the modified y6, y7, and y8 ion confirmed a mass increase of 1.0 Da. (TIFF 217 kb) [file 13075_2018_1562_MOESM4_ESM.tif]

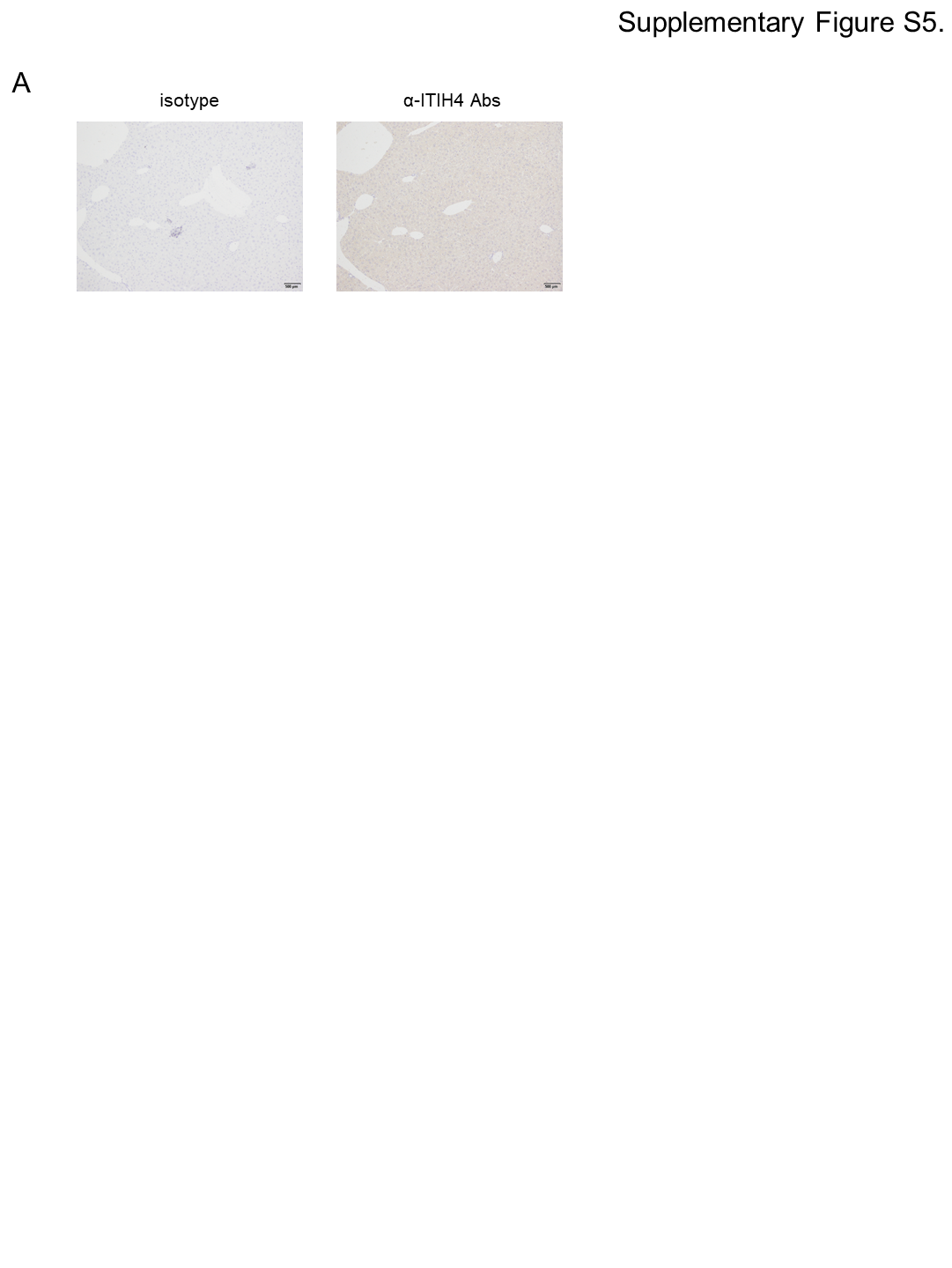

Supplement: Supplementary file 5 — Figure S5. (a) Liver tissue sections from naïve mice were immunohistochemically stained with anti-ITIH4 antibodies as a positive control to detect ITIH4. (TIFF 263 kb) [file 13075_2018_1562_MOESM5_ESM.tif]

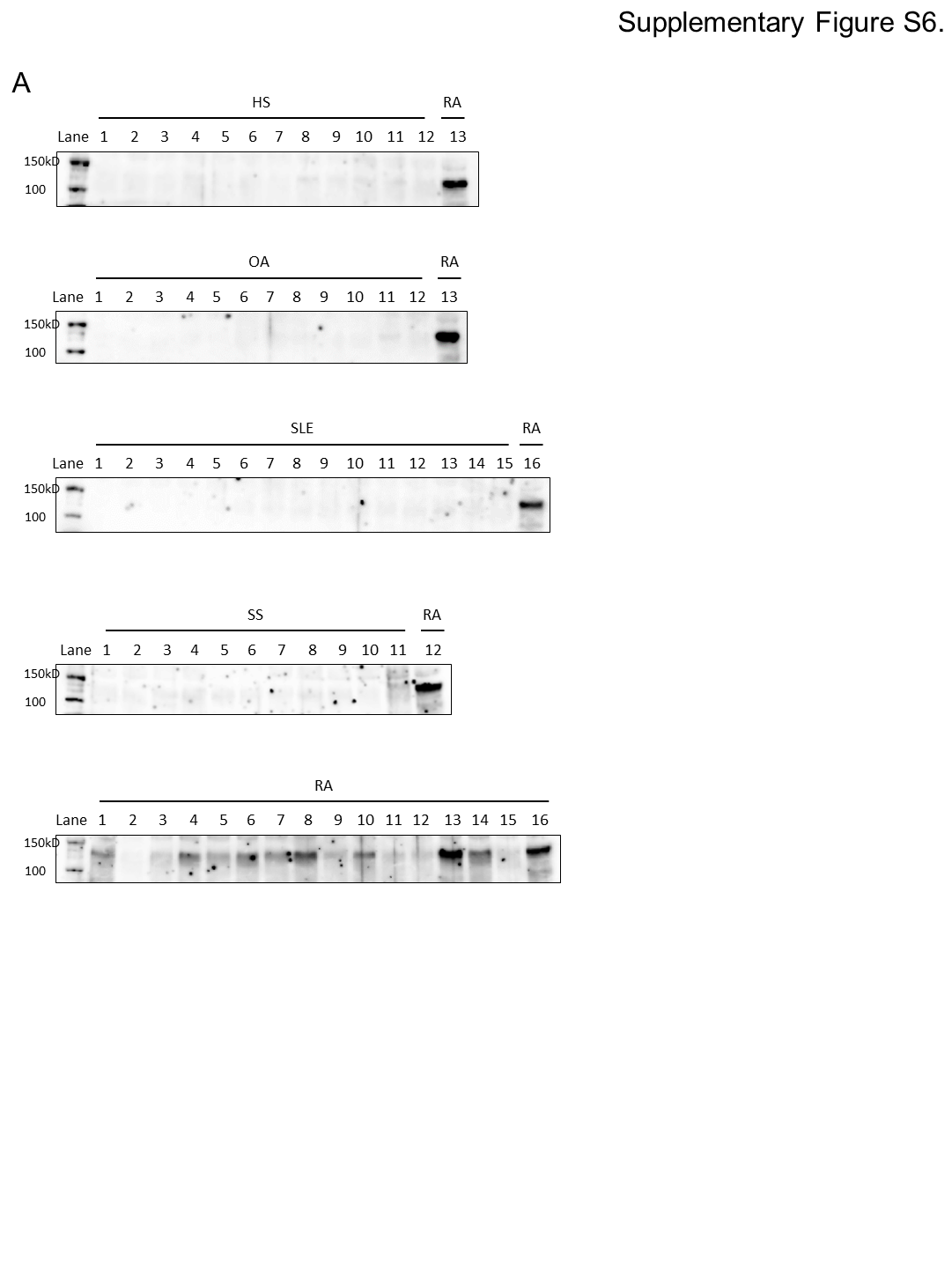

Supplement: Supplementary file 6 — Figure S6. (a) Sera from patients with RA, HS, patients with OA, patients with SLE, and patients with SS was subjected to Western blot analysis using AMC antibodies. Citrullinated proteins were specifically detected as an ~ 120 kDa band in patients with RA. (TIFF 238 kb) [file 13075_2018_1562_MOESM6_ESM.tif]
